# Patterns in Coral Fecundity Across the Indo‐Pacific

**DOI:** 10.1002/ece3.74441

**Published:** 2026-10-02

**Authors:** M. M. Bilodeau, K. M. Quigley

**Affiliations:** ^1^ College of Science and Engineering James Cook University Townsville Queensland Australia; ^2^ The Minderoo Foundation Perth Western Australia Australia; ^3^ University of Western Australia Perth Western Australia Australia

**Keywords:** colony fecundity, coral reefs, fecundity per cm^2^, reproduction, total fecundity

## Abstract

Coral reproductive potential is a fundamental component of reef resistance and recovery, yet large‐scale estimates of reproductive potential remain limited. This study generated the first large‐scale approximation of coral reproductive potential across the Indo‐Pacific by integrating coral fecundity, trait‐based imputation, and coral cover data. Reproductive potential was estimated for 277 coral species from 55 genera, including key genera such as *Acropora*, *Isopora*, *Montipora*, and *Porites*, for a single, hypothetical spawning period. We combined empirical coral fecundity datasets with trait‐based imputation approaches to estimate reproductive potential at three levels: fecundity per cm^2^, colony fecundity, and total fecundity across the Indo‐Pacific. These fecundity estimates were then integrated with regional coral cover to derive a first‐order approximation of reproductive potential. The estimated total reproductive potential of Indo‐Pacific corals was approximately three quintillion eggs (3.46 × 10^18^) for one hypothetical spawning period. Fecundity varied significantly across genera and growth forms, with *Isopora*, *Acropora*, and *Montipora* contributing disproportionately to total egg production (mean totals of 3.9 × 10^16^, 4.2 × 10^15^, and 3.49 × 10^15^ eggs, respectively). Branching and massive growth forms collectively accounted for roughly 50% of total fecundity across all levels. Species‐level fecundity was not consistently aligned with genus‐level trends, with some species exhibiting exceptionally high fecundity at the species level despite relatively low average fecundity at the level of genus. Concordance across the three fecundity levels was low, highlighting substantial variation within genera and potential gaps in our current understanding of coral reproductive potential, likely both. This study provides the first quantitative approximation of Indo‐Pacific coral reproductive potential, demonstrating high variation across species, genera, and growth forms. These findings underscore the importance of multi‐scale reproductive assessments and offer a data‐driven foundation for identifying key taxa for conservation or restoration efforts based on their disproportionately high contribution to reef recovery.

## Graphical abstract

This study provides the first large‐scale approximation of coral reproductive potential across the Indo‐Pacific by integrating empirical fecundity data, trait‐based imputation, and regional coral cover estimates for 277 species across 55 genera. Total reproductive output was estimated at 3.46 × 10^18^eggs for a single spawning event, with substantial variation among species, genera, and growth forms. These findings highlight major gaps in reproductive datasets and provide a quantitative framework for identifying key taxa that may disproportionately influence reef recovery and conservation outcomes.

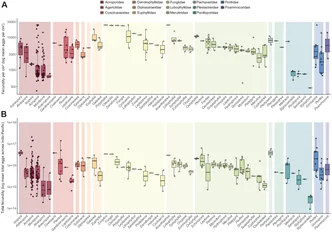

## 1 Introduction

Climate change is the most pressing concern for coral reefs globally, with ocean warming driving most coral cover decline in recent decades (De'ath et al. 2012; Hoegh‐Guldberg et al. 2007; Hughes et al. 2017). The Indo‐Pacific region hosts some of the world's richest coral biodiversity (Connolly et al. 2003). However, long‐term impacts of repeated disturbances on coral diversity and abundance remain uncertain. Certain species, particularly those within the genus *Acropora*, have shown considerable vulnerability to rising temperatures, leading to significant population oscillations through time (Hughes et al. 2010; Quigley et al. 2022). These disturbances disrupt the intricate ecological relationships that support reef biodiversity and productivity (Emslie et al. 2020; Pandolfi et al. 2005). It is therefore essential to pursue climate mitigation strategies and localized conservation efforts to preserve these vital ecosystems for future generations (Graham et al. 2014; Van Hooidonk et al. 2014).

Coral reproduction underpins reef recovery. This is critical given disturbances such as bleaching and mortality can decrease coral fecundity and therefore reproductive potential (Timmins‐Schiffman et al. 2024; Henley et al. 2021; Leinbach et al. 2021; Baird and Marshall 2002). Successful recovery after disturbance is dependent on factors like the timing of spawning events (Sorek and Levy 2014), extent and direction of larval dispersal (Thomson et al. 2021), and habitat availability for settlement (Doropoulos et al. 2015). Thus, studying reproductive processes can help explain both the drivers and variation of natural recovery as well as guide targeted conservation actions to bolster coral resilience (Gouezo et al. 2019). Reproductive success across multiple life‐history stages (e.g., fertilization success, larval survival, and successful settlement) is crucial for population resilience. Survival at each of these stages is required after disturbances to ensure a supply of individuals (either through long‐distance dispersal or through local recruitment) to maintain populations, and facilitate recovery (Edmunds 2023; Hughes et al. 2010; Wallace 1985). Fundamental knowledge around coral reproductive traits is critical to inform the ecology of reefs as well as conservation and management strategies. Tangible examples include guidance on the designation of protected areas as larval sources (Hock et al. 2017) and the timing of protections (i.e., cessation of dredging or extraction) to protect reproductive processes (Carter et al. 2017; Selig and Bruno 2010).

Quantifying coral reproductive potential is also essential for modeling population dynamics, forecasting population trends and assessing environmental impacts (Bramanti et al. 2015). Especially critical is understanding how climate‐related stressors, like temperature, may affect reproductive traits that underpin reef recovery after a disturbance. Given coral reproduction is highly variable across species (Harrison 2011; Foster and Gilmour 2020), and influenced by numerous biological and environmental factors (Keith et al. 2023; Van Woesik and Jordán‐Garza 2011), it is possible that other less well studied reproductive traits also vary across coral species (Shlesinger et al. 1998; Foster and Gilmour 2020; Henley et al. 2021). Important reproductive traits include: the total number of eggs produced (hereafter fecundity), oocyte size, fertilization success, larval settlement, and post‐settlement survival (Adjeroud et al. 2017; Beiring and Lasker 2000). Factors such as oocyte size can be beneficial for corals success as larger oocyte sizes can provide higher success rates for survivorship (Foster and Gilmour 2020), however this also comes with a trade‐off as larger oocyte sizes are more resource costly (Álvarez‐Noriega et al. 2016; Leuzinger et al. 2003). Coral fecundity varies considerably across species, depending on factors like colony size, age, and health (Hughes and Tanner 2000; Rapuano et al. 2023). Environmental factors, such as temperature, play a critical role in driving patterns in coral reproduction; where elevated temperatures that cause bleaching have been linked to reduced egg production (Baird and Marshall 2002; Jokiel and Coles 1990; Timmins‐Schiffman et al. 2024; Briggs et al. 2024). Life‐history strategies also vary across coral taxa, strongly influencing their responses to disturbances (Darling et al. 2012). Fast‐growing, broadcast‐spawning genera like *Acropora* are often the most heavily impacted by disturbances such as bleaching or cyclones but tend to recover rapidly due to high recruitment and growth rates (Morais et al. 2024). In contrast, slower‐growing, massive or encrusting taxa may show lower immediate mortality but exhibit limited recovery due to reduced recruitment and slower population turnover (Done and Potts 1992; Morais et al. 2021). As such, disturbance impacts that appear modest in terms of short‐term mortality may mask longer‐term shifts in community structure and reef function. Recognizing contrasting life‐history traits is critical for understanding and predicting coral reef resilience.

Coral reproduction represents a substantial energetic investment, and before spawning, corals may allocate approximately 20%–40% of protein and lipid resources into their reproductive tissues (Leuzinger et al. 2003; Leinbach et al. 2021). Reproductive success in corals has also been measured through oocyte size, which includes measurement of eggs using microscopy and staining (Rapuano et al. 2023), and generally, higher reproductive success has been equated to large oocyte size (Foster and Gilmour 2020). Environmental stress can alter the allocation of these resources, reducing overall gamete production and quality (Timmins‐Schiffman et al. 2024; Leinbach et al. 2021). Importantly, reproductive success is determined by more than fecundity (i.e., number of eggs). It also includes factoring in oocyte size and gamete quality, given these traits represent additional components of reproductive investment and can influence subsequent, overall reproductive success (Foster and Gilmour 2020; Gutierrez‐Isaza et al. 2023). Environmental conditions experienced by gametes can also influence fertilization success (Humanes et al. 2017; Ricardo et al. 2015), where post‐settlement survival ultimately determines whether successful fertilization and settlement translate into recruitment (Edmunds 2023). Consequently, colonies producing similar numbers of eggs may differ substantially in their realized contribution to recruitment. Finally, although there is evidence of spatial variation in egg size and resulting fecundity (Santacruz‐Castro 2019; Gutierrez‐Isaza et al. 2023), this variation has not been directly related for corals to temperature or latitude (Thorson‐Rass rule), as has been determined for other marine species (Gutierrez‐Isaza et al. 2023; Laptikhovsky 2006).

Quantifying the number of coral larvae produced across reefs is essential for understanding the reproductive capacity and resilience of coral populations. It allows for the identification of reefs or regions that may serve as major larval sources, sustaining regional connectivity and facilitating recovery after disturbances. This information underpins effective management and restoration strategies by revealing where protection or intervention will have the greatest impact on replenishing degraded reefs. This work also highlights critical gaps in information around spawning observations—including the timing, frequency, and size of colonies participating in spawning events—which are essential for accurately quantifying reproductive output and connectivity.

Here, we calculated estimates for coral fecundity at three levels (per cm^2^, colony, and Indo‐Pacific) across hundreds of coral species. Using coral cover data from the Indo‐Pacific region, estimates of total reproductive potential are presented. These analyses address significant gaps in estimates of coral fecundity and show significant variation across coral species, genera, and growth forms. This study provides first‐order approximations of coral fecundity across the Indo‐Pacific by combining existing coral trait data and imputation methods across multiple levels. This provides baseline estimates of how coral fecundity varies across taxa and reef regions—information that is important for guiding conservation decisions on what taxa and locations are vulnerable (i.e., may have lower fecundity). It also provides data for the development of predictive models of reef resilience under on‐going climate change impacts.

## 2 Methods

### 2.1 Defining Coral Reproductive Traits and Individual Colony Fecundity

Fecundity refers to the reproductive potential of an organism or population, typically measured as the number of gametes (eggs or sperm concentration), seeds, or asexual propagules an individual or group produces over a given time‐period (Bradshaw and McMahon 2008). Coral fecundity has been quantified by counting the gametes (egg/sperm bundles or planula) produced per polyp or per colony (Richmond 1997). In this study, we define fecundity as the total estimated number of eggs produced (Bradshaw and McMahon 2008). Hence, this metric does not incorporate oocyte size or quality, fertilization or settlement success, or subsequent survival. It should therefore not be interpreted as total realized reproductive success.

We obtained trait data for 277 coral species across 35 fecundity related traits from the Coral Traits Database (Madin et al. 2016). The following traits were sourced from the Coral Trait Database, in which we list the trait number (as designated in the Database) with its corresponding number of empirically measured observations (*n* = number of observations). The traits that were later used for imputation (see below) were as follows: colony planar area (Coral Trait 155, measured in mm^2^ and then converted to cm^2^, *n* = 1520 entries across 9 species), polyp per area (Coral Trait 148, polyp density per projected planar area of colony, measured in cm^2^, *n* = 36 entries across 14 species), polyp fecundity (Coral Trait 12, number of eggs per polyp, *n* = 44 entries across 10 species), and colony area (Coral Trait 155 (and 57), colony area—as a proxy for size—is the planar area of the colony in cm^2^, *n* = 1520 entries across nine species). The final dataset of traits comprised 277 coral species from 55 genera. Some traits were not available for all species. Empirical fecundity measurements were retained where available. Trait‐based imputation was therefore used to estimate missing fecundity trait values, allowing reproductive potential to be approximated across species for which direct measurements were unavailable. For example, only nine species had empirical colony‐area measurements available; meaning colony‐area measurements for other species were subsequently estimated through the trait‐imputation procedure.

Average colony size was determined per species using colony area, in which nine species were available; for the remaining species, colony area was imputed following methods outlined above. This is an important simplifying assumption, where we know that fecundity can scale with colony size for different species (Álvarez‐Noriega et al. 2016), however, due to lack of data, we are applying an average colony size per species. Here, fecundity was assessed across multiple hierarchical levels. The smallest level was calculated at the polyp‐level (fecundity per cm^2^), defined as the number of eggs produced per square centimeter of coral tissue. To calculate fecundity per cm^2^, polyp per area was multiplied by polyp fecundity. Colony‐level fecundity was then estimated (mean number of eggs per colony, normalized to the average‐sized colony size). This was defined as the total number of eggs produced by a single average‐sized colony per species. To calculate colony fecundity, average colony size was multiplied by fecundity per cm^2^. Total fecundity was defined as the total number of eggs per species across the Indo‐Pacific. This value was calculated by multiplying the fecundity per cm^2^ by the percent cover of each species by total geographic distribution of that species in km^2^.

To achieve general estimates of fecundity across a spatial level, fecundity was linked with coral cover. To link fecundity to coral cover, we have made some simplifying assumptions. First, we assume that the number of eggs produced per polyp (polyp‐level) is held constant as the colony ages/grows. Although we acknowledge the size‐fecundity relationship in corals is not generally linear (Hall and Hughes 1996), and there is variation in this relationship across at least 8 species (Álvarez‐Noriega et al. 2016), we assume that the calculation for an average‐size colony should result in an average estimate of fecundity, not a minimum or maximum based on a range of size estimates. Second, we assume that a majority of species across the Indo‐Pacific are undergoing reproduction during a single hypothetical spawning period. Therefore, this data should not be interpreted as the total cumulative annual reproductive potential for all species. Species and populations vary in reproductive timing and frequency, including some species that undergo biannual spawning, asynchronous reproduction or even monthly planulation (Bouwmeester et al. 2021; Osman et al. 2024; Foster and Gilmour 2020). However, we have limited these calculations to a single reproductive event as most species undertake broadcast spawning once per year (Gilmour et al. 2016). Our third assumption is that an average colony size represents an average value within a distribution of colony size frequencies, given the caveat that colony size frequency distributions are skewed (Bak and Meesters 1998). The vast majority of colonies are generally small and below the size threshold of sexual maturity (Bak and Meesters 1998) and this ratio varies widely between species and depends on size at maturity and the shape of the size frequency distribution (Bak and Meesters 1998). Given the paucity of reproductive data for most coral species, especially over time, these two relationships may cancel each other out, but these remain major assumptions of this work. In summary, although it is likely that only a few colonies contribute disproportionately to reproductive potential and success of their species, given reproductive potential scales exponentially with colony size and that most colonies are too small to reproduce at all. By using an “average‐colony size” estimate, we provide a first‐order approximation with clear assumptions, and strongly recommend further work to validate these estimates.

### 2.2 Extrapolating to Indo‐Pacific Estimates Using Coral Cover

The total area of coral cover per species across the Indo‐Pacific was sourced from the Status of Coral Reefs of the World: 2020, with an estimated mean coral cover of Indo‐Pacific equaling ~189,498 km^2^ (Souter et al. 2021). To estimate coral fecundity per species across the Indo‐Pacific region, we used species‐specific range sizes (Dietzel et al. 2021). Species distribution data were sourced from Dietzel et al. (2021) and coral cover from Souter et al. (2021), as explained above.

To calculate total fecundity, estimates of mean coral cover for each species were multiplied by their distribution (i.e., total area) across the Indo‐Pacific and then multiplied by each species to get estimated fecundity potential. To determine total reproductive potential across the Indo‐Pacific reefs, the total average fecundity across species was summed together and then divided by the total number of species. This resulted in an estimated total average reproductive potential (average number of eggs produced by coral across Indo‐Pacific coral reefs during a single hypothetical spawning period).

### 2.3 Imputation of Missing Fecundity Values

Data on coral fecundity is limited for many species, including in the Indo‐Pacific. For the > 600 coral species recorded in the Indo‐Pacific (Dietzel et al. 2021), data for only 277 species are available for this region from the Coral Traits Database (Madin et al. 2016), which included direct measured fecundity data for only 10 of these species. The R package “funspace” (Carmona et al. 2024) was therefore used to impute missing data for these other species. This package employs trait‐based data imputation and ordination methods using Principal Components Analysis (PCA) to estimate missing trait values. Fecundity estimates for most of the remaining species were estimated. All fecundity calculations were initially performed at the species level for the 277 species included in the final traits dataset. Species‐level estimates were then subsequently aggregated to the level of genus as well as by growth form. This allowed us to examine broader taxonomic and morphological patterns in reproductive potential. Therefore, all calculations were performed at the species level, including colony area, polyp density, polyp fecundity, and resulting colony fecundity. Thus, calculations at the level of genus did not assume a uniform colony size or number of eggs; instead, it was calculated for each species individually. Further, in assessments of how well imputation performed, known empirical values were checked against imputed estimates. To do this, empirical colony fecundity (number of eggs per cm^2^, Coral Trait 216, *n* = 203 entries, *n* = 6 species) was downloaded from the Coral Trait Database. We found that our estimated values followed the same general trend, although with a tendency to overestimate, mostly around very large fecundity estimates (see Figure S1).

### 2.4 Statistical Analysis

All statistical analyses were performed in R (version 2024.12.1). Statistical comparisons of fecundity were conducted separately from the spatial extrapolation of reproductive potential. Fecundity estimates were first evaluated for variation among species, genera, and growth forms. Species‐specific fecundity estimates were then combined with coral cover and geographic distribution to estimate reproductive potential across the Indo‐Pacific. To evaluate baseline variation in fecundity prior to spatial extrapolation, we used Analysis of Variance (ANOVA) to test for differences in per‐unit fecundity estimates across taxonomic groups (species and genera), spatial scales (locations), and morphological traits (growth forms). Growth form categories were defined following Dietzel et al. (2021) and included: massive, branching (combined open and closed branching), laminar, encrusting, digitate, columnar, corymbose, encrusting long uprights, hispidose, sub‐massive, and tables/plates.

The percentage of total fecundity attributed to each species, genus, and growth form was calculated at the level of cm^2^, colony and total fecundity. Given seven genera accounted for > 50% of the reproductive potential estimated in our dataset, these seven were then used as the focus for our comparative analysis. The relative reproductive potential, expressed as a percentage of the total reproductive potential at each fecundity level, was calculated to determine if any species, genera, and growth forms explained a disproportionate amount of variation in reproductive potential. A three‐way ANOVA was used to examine any significant differences in variation across species, genera, and growth forms. The remaining genera not included in the top seven were classified as “other” and included the other 49 genera. For growth form, “other” refers to six growth forms (columnar, corymbose, encrusting, hispidose, sub‐massive, and tables/plates).

## 3 Results

### 3.1 Fecundity Estimates Across Multiple Spatial Levels

Across all levels of fecundity measured here, estimated fecundity varied significantly across genera. This includes across eggs per cm^2^ (hereafter referred to as fecundity per cm^2^), colony fecundity (average total eggs per colony), and total fecundity (total eggs produced across the Indo‐Pacific) (Figure 1A–C).

**FIGURE 1:**
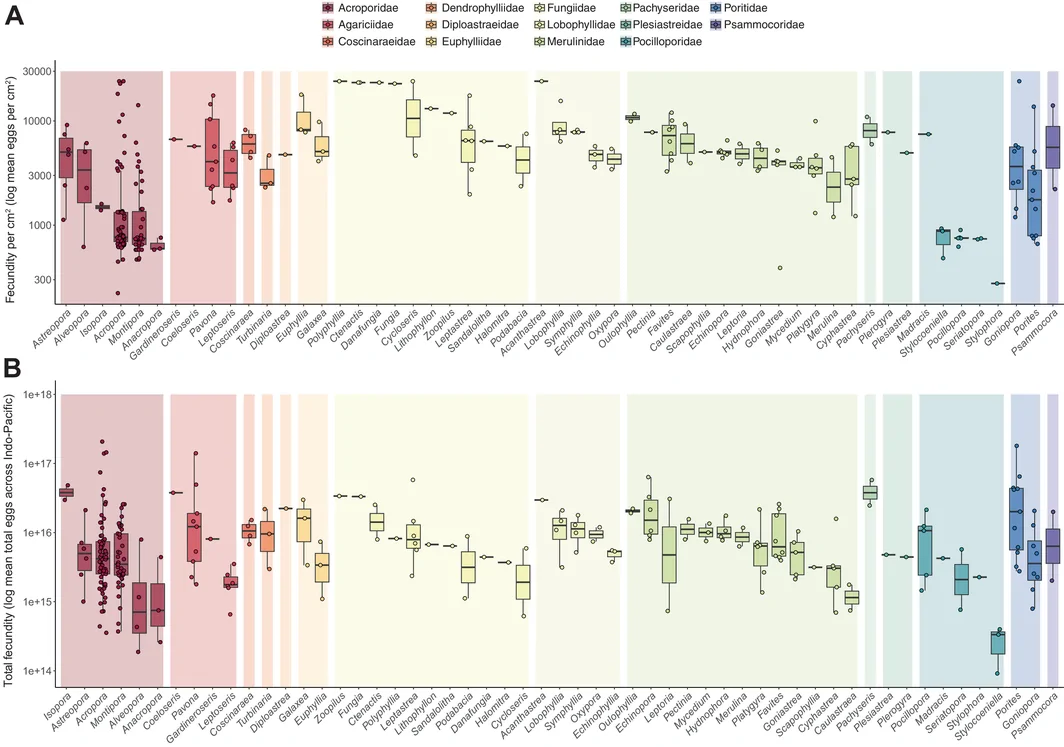
Comparison of fecundity estimates across coral genera and families. Colors correspond to family and are consistent for plots A and B. (A) Mean fecundity per cm^2^ per genera ranked from highest to lowest. (B) Mean total fecundity per genera estimated across the Indo‐Pacific, from highest to lowest median value.

Across all genera, fecundity per cm^2^ ranged from 276 eggs/cm^2^ (*Stylophora*) to 24,046 eggs/cm^2^ (*Polyphyllia*), with an average of 6877 eggs/cm^2^ (±826 SE). Fecundity per cm^2^ was significantly different between genera (*p* = 3.69 × 10^−15^). After *Polyphyllia*, other Fungiidae like *Ctenactis* (23,353 eggs/cm^2^) and *Danafungia* (23,350 eggs/cm^2^) recorded the highest values (Figure 1A).

Total fecundity, defined as the total number of eggs produced across the Indo‐Pacific, was estimated to be 3 quintillion eggs (3.46 × 10^18^) during a single hypothetical spawning period across all 277 coral species examined here. Compared to fecundity per cm^2^ and colony fecundity, total fecundity followed a different pattern. *Pachyseris* (4.12 × 10^16^ eggs), *Isopora* (3.9 × 10^16^ eggs), and *Coeloseris* (3.77 × 10^16^ eggs) recorded the highest total fecundity (Figure 1B). Total fecundity ranged from 3.34 × 10^14^ eggs (*Stylocoeniella*) to 4.12 × 10^16^ eggs per km^2^ (*Pachyseris*), with an average of 1.09 × 10^16^ eggs per km^2^ across all genera (±1.39 × 10^15^ SE). Differences in total fecundity did not vary significantly across genera (*p* = 0.959); however, did vary significantly between levels of fecundity and genera (*p* = 1.76 × 10^−4^).

The smallest level of fecundity (fecundity per cm^2^) did not correlate strongly with the largest estimate of fecundity (total fecundity) across any of the species measured (*R* ^2^ = 0.217, *p =* 2.46 × 10^16^, Figure S3, Table S1). These two measures also did not correlate equally across growth forms, including branching and digitate corals (*R* ^2^ = 0.08–0.713, *p =* 1.26 × 10^−14^–0.111). The only exceptions were for the laminar, encrusting and massive growth forms, where there was a significant correlation between fecundity per cm^2^ and total fecundity (*R* ^2^ = 0.212, 0.08, and 0.09, *p =* 0.005, *p =* 0.111, and *p* = 0.0121). Overall, fecundity per cm^2^ was weakly correlated to total fecundity (Figure 2).

**FIGURE 2:**
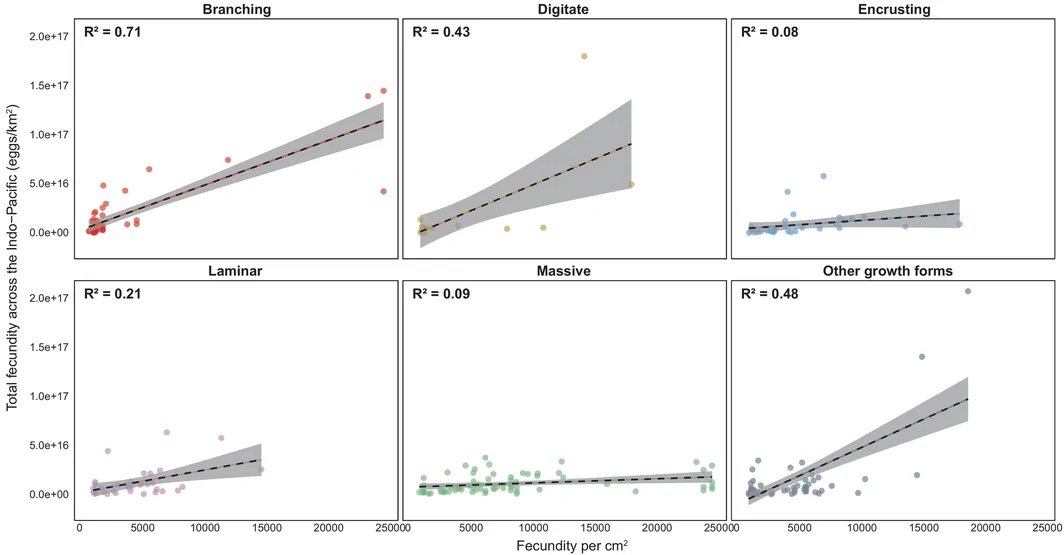
The relationship between fecundity per cm^2^ and total fecundity estimated across the Indo‐Pacific by growth form. Colors represent growth forms, where each point is a different coral species within that growth form. The “other” category represents the remaining growth forms that contribute to the total reproductive potential (see Section 2). Dashed lines represent linear regression and shaded bands represent standard errors.

Colony fecundity, measured as the total number of eggs produced per average colony size, ranged between a mean of 34,040 eggs per colony (*Stylophora*) to 3,744,314 eggs (*Polyphyllia*), with a mean of 1,130,959 eggs per colony across all species (±121,113 SE) (Figure S2). Similar to estimates of fecundity per cm^2^, the highest colony fecundity estimates were estimated in *Polyphillia* (3,744,314 eggs), *Acanthastrea* (3,744,314), *Danafungia* (3,644,423), and *Ctenactis* (3,577,421) (Figure S2). Differences in colony fecundity across genera were also statistically significant (*p =* 1.97 × 10^−7^).

### 3.2 Total Reproductive Potential Across Different Coral Genera and Growth Forms

Total reproductive potential in the five most fecund genera was examined (Figure 3A). Here, total reproductive potential is defined as the percentage of eggs (out of 100%) produced per genera or per growth form at each of the three fecundity levels examined: fecundity per cm^2^, fecundity per colony, and total fecundity across the Indo‐Pacific. Potentials differed significantly across the genera and fecundity levels (ANOVA: per cm^2^ *p =* 4.956 × 10^−4^, colony *p =* 9.96 × 10^−4^, Indo‐Pacific *p* = 1.82 × 10^−3^, between fecundity levels *p =* 1.77 × 10^−4^) (Figure 2A). Across all three levels, *Acropora* contributed to the greatest potential (15.1%, 22.6% and 28.6% for per cm^2^, per colony and total, respectively), followed by *Montipora* or *Porites* (5.47%, 8.05% and 12.23%, per cm^2^, per colony and total Indo‐Pacific, respectively for *Montipora*). When examined at the scale of fecundity per cm^2^, total reproductive potential varied significantly between genera (*p =* 1.77 × 10^−4^). Moreover, 37.9% ± 0.018 (SE) of total reproductive potential was attributed to these top five genera, with again, *Acropora* having the highest reproductive potential (15.1%) (Figure 2A). The remaining 62.1% ± 0.002 (SE) (“other growth forms”) was made up of the remaining genera which all had a range of relative reproductive potentials from 3.5% to 0.11%.

**FIGURE 3:**
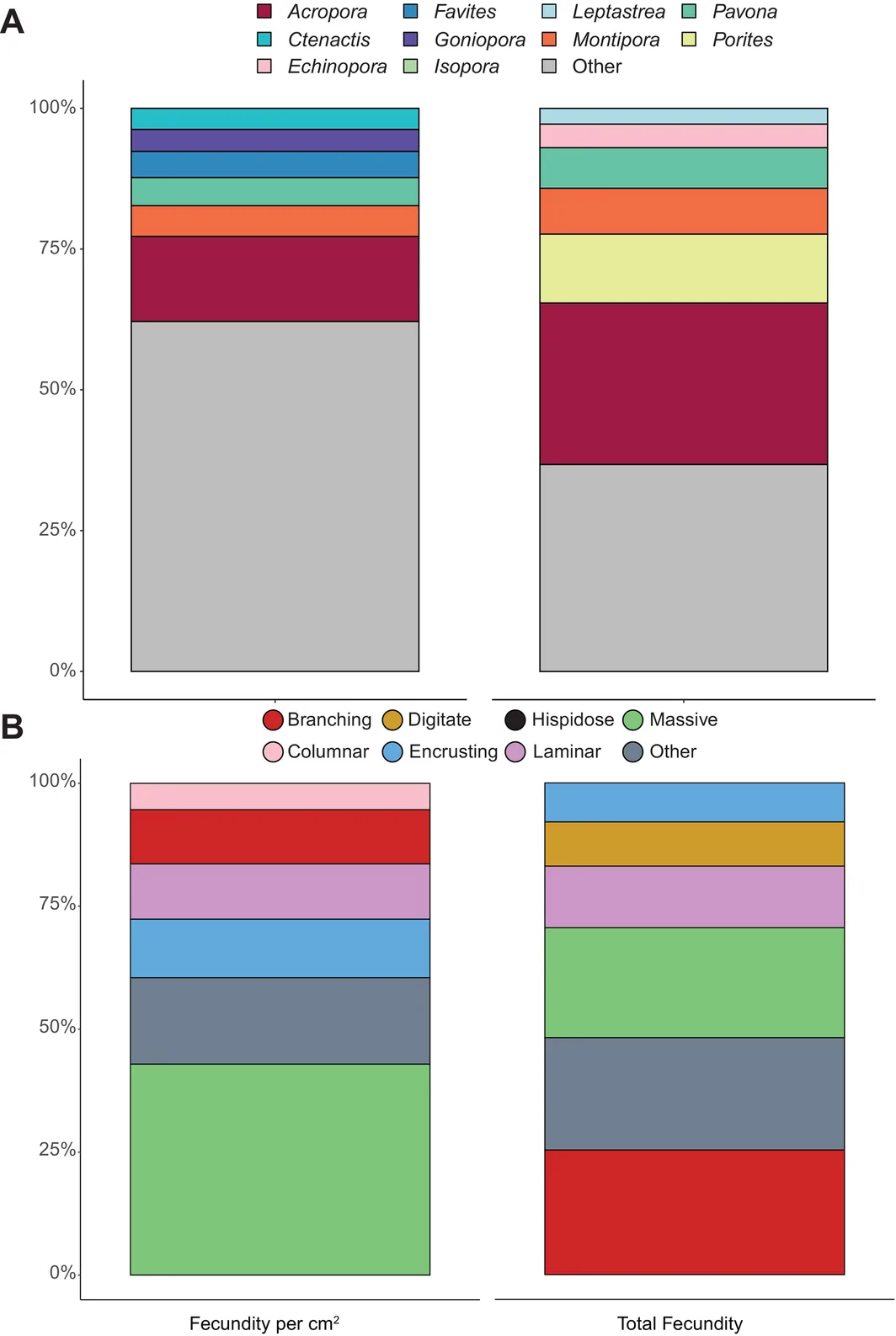
Proportion of total reproductive potential across selected by (A) coral genera and (B) growth forms. The proportion of total reproductive potential for six of the major genera, as well as the remaining genera (“other”) fecundity per cm^2^ and total fecundity. Each genus or growth form is represented by a different color.

Colony fecundity followed a similar pattern, in which the five most abundant genera (*Acropora*, *Montipora*, *Porites*, *Favites*, and *Pavona*) made up 45.6% ± 0.031 SE of the total reproductive potential (23%, 8%, 4.2%, 3.7%, and 3.7% respectively). Conversely, over 63.2% ± 0.039 (SE) of total reproductive potential across the Indo‐Pacific was attributable to the top five genera (28.6% *Acropora*, 12.2% *Porites*, 8.1% *Montipora*, 7.2% *Pavona*, and 4.2% *Echinopora*).

Of the top five genera with the highest fecundity, three genera had both the highest fecundity per cm^2^ as well as the highest total fecundity. These included *Acropora* (15.1% per cm^2^ fecundity, 28.67% total fecundity, respectively), *Montipora* (5.47%, 8.15%) and *Pavona* (4.98%, 7.2%). Other genera such as *Isopora* and *Pocillopora* demonstrated a higher total reproductive potential across the Indo‐Pacific (2.3% or 7.8 × 10^16^ and 1.4% or 4.83 × 10^16^ total eggs across the Indo‐Pacific, respectively). However, fecundity for these genera, at per cm^2^ and colony fecundity was relatively low, with *Isopora* producing 2993 eggs/cm^2^ (0.24% of the total reproductive potential at that scale) and 1,613,646 eggs/colony (0.66%). *Pocillopora* produced 3781 eggs/cm^2^ (0.30%) and 1,834,087 eggs/colony (0.75%) (Figure 2A). In contrast, *Favites* exhibited high fecundity at the per cm^2^ and per colony levels, producing 57,802 eggs/cm^2^ (4.65%) and 9,117,005 eggs/colony (3.73%), but contributed relatively little to total reproductive potential across the Indo‐Pacific (2.64%, 9.14 × 10^16^ eggs, Figure 2A).

The three levels of fecundity were also examined for each growth form (Figure 3B). Massive and branching forms made up ~50% of the total reproductive potential across the three levels. Massive growth forms had the highest total per cm^2^ and colony fecundity (42.89% and 36.45%, respectively), whereas branching growth forms were the most fecund across the Indo‐Pacific (25.34%). The proportion of total reproductive potential varied significantly across fecundity levels for growth form at per cm^2^ and colony levels (ANOVA: *p =* 6.98 × 10^−6^ and *p =* 0.00959), but not for total fecundity (*p* = 0.253). Across growth forms, reproductive potential also varied significantly between the three fecundity levels (*p* = 0.032).

Species within the same genus differed significantly in estimates of fecundity per cm^2^ (Figure 4, Figure S4, *p* = 3.69 × 10^−15^). Within *Acropora*, the mean fecundity per cm^2^ was 2642 ± 0.039 (SE) eggs but ranged widely across species, including from 233 eggs per cm^2^ ( *Acropora nana* ) to 2642 eggs per cm^2^ (*Acropora fastigata* and *Acropora insignis* ). Similarly, *Montipora* and *Porites* displayed substantial variation, from 468–14,170 eggs per cm^2^ (mean 1618 eggs per cm^2^, lowest: *M. altasepta* and *M. corbettensis* , highest: *M. verrucosa* , Figure 3) and 661–13,713 (mean 3082 eggs per cm^2^, lowest: *P. rugosa* , highest: *P. rus* ).

**FIGURE 4:**
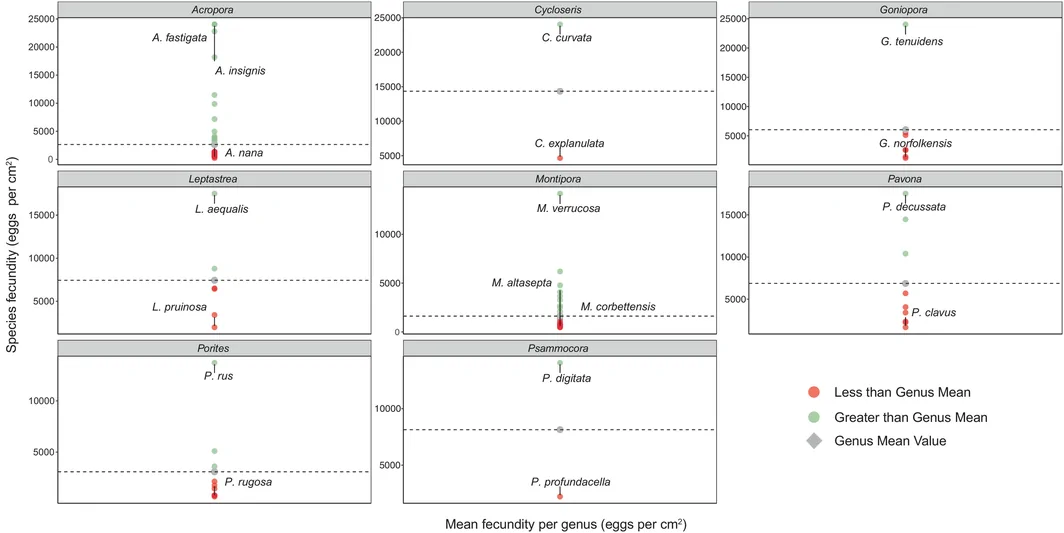
Mean fecundity per cm^2^ per species relative to mean fecundity per genus. Gray dots and the dashed black lines represent the mean fecundity per cm^2^ per genus. Species with a mean value greater than the mean per genus are colored in green and those below the average are colored in red.

## 4 Discussion

### 4.1 Estimated Coral Fecundity Across the Indo‐Pacific

Ecologists often seek to understand the quantity of things. Whether that is the number of ants (Schultheiss et al. 2022), trees (Sagar and Singh 2005), or corals (Dietzel et al. 2021), abundance can reveal critical insights into how ecosystems function and their health. To that end, we estimated coral fecundity across multiple levels, ranging from the individual polyp (cm^2^) to across 189,498 km^2^ of coral cover in the Indo‐Pacific.

Scleractinian corals, and by extension, coral reefs, are known for their high reproductive potential due to many species undergoing mass spawning (Sorek and Levy 2014). This massive potential is reflected in the first‐order estimate of total fecundity derived here, at approximately 3 quintillion eggs (3.46 × 10^18^). This underscores the immense reproductive potential, and therefore the regenerative capacity, of these ecosystems. This estimated potential production of eggs across the Indo‐Pacific in a single hypothetical spawning period highlights the crucial role these reefs play in global marine biodiversity by supplying the region with biomass and offspring across hundreds of species (Sorek and Levy 2014).

We emphasize that these values represent first‐order approximations and that due to the paucity in empirical data, the imputation of data was extensive and highlight the danger in overgeneralization and infilling of trait data (Kim et al. 2023). We recognize, therefore, that the overall values likely do not represent the “true” values, but only broad estimates.

Comparisons between brooding and broadcast‐spawning corals should also be interpreted cautiously because these two reproductive strategies differ in their fecundity traits (including gamete quality and number), as well as the timing and frequency of reproduction within each group (Foster and Gilmour 2020; Gilmour et al. 2016). Importantly, broadcast spawning species generally release very large numbers of gametes into the water during relatively few and discrete spawning events. In contrast, brooding species may release developed planulae over multiple reproductive periods per year, generally at lower abundance compared to broadcasting species (Shlesinger et al. 1998; Gilmour et al. 2016). In addition, reproductive mode is not necessarily conserved within coral genera, with genera such as *Pocillopora* and *Porites* containing both brooding and broadcast spawning species (Schmidt‐Roach et al. 2012). Reproductive mode may also vary geographically within some species, further demonstrating that broad genus‐level classifications can obscure important species‐ and population‐level variation (Combosch and Vollmer 2013; Schmidt‐Roach et al. 2012). In particular, 6 of the 277 total species included in this dataset are brooders that release planula larvae multiple times a year, although here we only estimated a single release period. This likely underestimates the contribution of brooders relative to spawners in our first‐order estimates. However, this underestimate may be relatively minor given previous work with these 6 species (*Isopora palifera*, *I. brueggemanni*, *Stylophora pistillata* , *Seriatopora hystrix* , *S. caliendrum, Euphyllia glabrescens*), which show similar larval release periods that align to spawning species (Foster and Gilmour 2018; Goodbody‐Gringley and De Putron 2016; Villanueva et al. 2008). Further work will require additional refinement of these estimates due to different reproductive modes. Indeed, crucial progress has been made to estimate these fundamental values, including methods that allow for reconstruction of colony size from line intercept transect data that would then facilitate estimates of community‐wide reproductive potential (Dietzel et al. 2020).

*Acropora*, *Porites*, and *Montipora* collectively contributed approximately 49% of the total estimated reproductive potential across the Indo‐Pacific, accounting for 9.92 × 10^17^, 4.23 × 10^17^, and 2.82 × 10^17^ eggs, respectively. In comparison with estimates derived from empirical studies of fecundity, however, these estimates, largely based on imputed values, may be underestimated. For *Acropora*, empirical measurements across seven species within this genus ranged from approximately 4–12 eggs per polyp (Foster and Gilmour 2020). Using imputed data, we found the median number of eggs per polyp to be approximately eight, within the range of empirical data. In contrast, the imputed data for *Montipora* was higher compared to empirical values (286,000 eggs vs. 100,000 eggs for a 250 cm^2^ colony of equivalent size, Henley et al. 2021; Henley et al. 2022). Finally, empirical fecundity measurements for *Porites* were highly variable, likely complicated by differences across species, locations, and over time. For example, different *Porites* species have been reported to produce approximately 40–100 to 5000 eggs cm^−2^ year^−1^ (Glynn et al. 2017). In comparison, the median imputed fecundity for the 11 *Porites* species included in our dataset was approximately 1754 eggs cm^−2^, which falls within the range reported. Together, these comparisons suggest that the imputation can approximate fecundity for some taxa, such as *Acropora* and *Porites*, but may over‐ or under‐estimate fecundity in other genera or species. This reinforces the importance of gathering additional empirical fecundity estimates and highlights that these values presented here represent the first‐order estimates of reproductive potential rather than precise predictions of fecundity.

This work represents the first estimate of semi‐global fecundity for corals with a focus on the Indo‐Pacific, the most biodiverse coral reef region globally. Global and regional analyses of fish fecundity in marine systems reveal that fecundity is driven by body mass of individuals (Hadj‐Hammou et al. 2024), although here, per colony fecundity did not scale to regional fecundity and instead is driven more by total abundance. Regardless of the ultimate drivers, this high reproductive potential is essential for sustaining coral populations and providing resilience against disturbances such as bleaching, cyclones, or Crown of Thorns outbreaks. Moreover, this work highlights the knowledge gaps in reproduction in coral reef research and serves to invigorate the effort on collection of this critical data, including egg numbers, egg sizes, and colony size‐frequency distributions. This work should represent the first, not the last, effort in understanding small‐ and large‐scale patterns in reproduction for corals, even if progress in this field has slowed due to the difficulty in understanding these relatively rare phenomena like coral spawning across such a speciose group of taxa.

Notably, we found genera with high fecundity, such as *Acropora*, *Montipora*, and *Porites*, contribute greatly to Indo‐Pacific fecundity despite not having the highest polyp or colony fecundity. Although, overall, *Acropora*, *Montipora*, and *Porites* all measured consistently high fecundity across the three spatial levels (per cm^2^, colony, and Indo‐Pacific). This finding is consistent with previous studies, which note that these genera often dominate reef‐building communities (Moritz et al. 2021), likely due to their relatively prolific reproductive output (Foster and Gilmour 2020; Holstein et al. 2016; Padilla‐Gamiño et al. 2013). This output may be one of the underlying drivers of their dominance. This reproductive output may also contribute to their rapid population recovery following disturbances and reinforces their role as keystone reef builders and maintainers of reef structure (Quigley and Baird 2024). Furthermore, this variation in output across genera may help explain differences in local abundances and underscores the importance of species‐ and genus‐level reproductive assessments to determine prediction metrics of reef resilience under environmental stressors.

### 4.2 Scale‐Specific Differences in Reproductive Potential

Patterns in fecundity varied across spatial levels. At local levels (per cm^2^ and colony), fecundity was dominated by a diversity of genera, not just *Acropora*. However, once scaled to the Indo‐Pacific, total fecundity was dominated by *Acropora*. This pattern was mostly driven by taxonomic diversity, and when assessed by growth form, variation in fecundity was mostly made up of massive species at smaller spatial levels. Genera like *Porites*, despite their low per cm^2^ fecundity estimates, also dominated Indo‐Pacific fecundity estimates. This could be due to their extensive geographic ranges or their greater abundance across that range relative to other species (Dietzel et al. 2021). This highlights the complex interplay between localized reproductive output and broader spatial and abundance distributions in determining overall reproductive potential (Fountain et al. 2019). These patterns underscore the level‐specific differences in assessing coral reproductive potential.

Variation in fecundity at small and large spatial levels suggests reproductive potential is driven by both the number of eggs produced and adult coral abundance. The incongruence between fecundity at small compared to large spatial levels supports evidence that total reproductive potential is not solely a function of fecundity at smaller levels (per cm^2^ and colony), but instead may better reflect other ecological factors like abundance (Sarribouette et al. 2022). This has often been discussed in terms of stock‐recruitment relationships and attempts to link sexually mature coral abundances with resulting offspring on the reef (Doropoulos et al. 2015). This has also included instances where local adult abundances do not scale to the number of offspring on the reef (Sarribouette et al. 2022). Our results may help to explain where some of this variation is originating and suggest variation in fecundity may exacerbate this overall stock‐recruitment mismatch. We also note that reproductive success includes other important factors not considered here, like egg size and egg or larval quality (Foster and Gilmour 2018; Padilla‐Gamiño et al. 2013) as well as other important demographic factors that influence dispersal success and survivorship (Figueiredo et al. 2024). Therefore, we do not assume that this preliminary assessment includes an exhaustive list of all relative factors.

Finally, disturbance and mortality may favor coral taxa with reproductive strategies that facilitate rapid recruitment. This may favor brooding species within the genus *Pocillopora* compared to spawning species within *Acropora* (Courtney et al. 2020). Combined with our results showing that brooders like some species of *Isopora* already dominate Indo‐Pacific reefs in terms of fecundity, this suggests that climate change will strengthen their dominance and potentially further accelerate shifts away from spawning (*Acropora*) to brooding (*Pocillopora*) species. These results align with studies emphasizing the necessity of both small‐ and large‐scale fecundity assessments for understanding coral reproductive dynamics and informing conservation strategies (Dietzel et al. 2021; Treml et al. 2012). It also highlights that regional dynamics are further complicated by other factors such as habitat availability and connectivity.

### 4.3 Relationship Between Fecundity and Coral Growth Form

There were significant differences between growth forms and two of the three levels of fecundity. Given the foundational empirical work by Hall and Hughes (1996) and Álvarez‐Noriega et al. (2016) across 6–8 species, our colony‐level fecundity estimates merely attempt to add to these empirical estimates by estimating across other, less empirically trackable species. When compared to these empirical estimates, we found that our estimates (~10^6^ eggs per colony) were within the estimated ranges, including for tabular (~10^3.5^–10^7^ eggs per colony) and corymbose/digitate *Acropora* (~10^3^–10^6^) or massive *Goniastrea* (~10^2.5^–10^6^ eggs per colony) (Álvarez‐Noriega et al. 2016). Moreover, since our estimates do not consider the influence of colony size completely (see “assumptions” in Section 2) and that the trait infilling method relies on sparse data, it is important to underscore that our estimates only represent first‐order approximations.

However, these empirical results do allow us to contextualize patterns here. For example, we expected massive species to have the highest per‐polyp fecundity, followed by corymbose, digitate, and tabular corals (Álvarez‐Noriega et al. 2016) and that colony‐level fecundity varied within genera and species (Hall and Hughes 1996). We also expected branching growth forms to dominate at both local and Indo‐Pacific scales due to their wider distributions and greater settlement success rates compared to other species (Vercelloni et al. 2024). Indeed, we found that fecundity significantly varied with growth forms across various fecundity levels tested. This included both massive and branching growth forms, which contribute disproportionately more to the variation across patterns in reproductive potential (Álvarez‐Noriega et al. 2016). Massive colonies have the capacity to grow to very large sizes and can support increased polyp density due to enhanced surface area for gamete production (Soong 1993). Alternatively, branching growth forms benefit from their rapid growth rates and high abundance (Dietzel et al. 2021; Roff 2020). This is reflected in their increased fecundity at the largest spatial level despite having relatively lower reproductive potential at the smaller spatial levels. These traits likely reflect trade‐offs between growth and fecundity and highlight the potentially complex relationship between morphology and reproduction.

### 4.4 Implications for Reef Resilience and Conservation

Whilst we report Indo‐Pacific fecundity on the order of quintillion, it is unclear how it compares to fecundity estimates before the beginning of mass bleaching events in 1999 (Goreau et al. 2000) or in relation to other reef regions. It is also important to note that coral cover estimates were collected between 1997 and 2006 and are best described as providing a baseline estimate of fecundity patterns ~20 years ago, before the occurrence of repeatedly severe marine heatwaves. As the most speciose reef region on the planet (Bellwood et al. 2005), this estimate may represent the maximum global reef reproductive potential. However, it is likely that this reproductive potential may not be sufficient to counteract the rapid and extensive degradation caused by climate change, which, along with other pressures, has led to significant declines in coral recruitment in the Caribbean and on the Great Barrier Reef (Hughes and Tanner 2000; Ortiz et al. 2018). The Indo‐Pacific has suffered repeated decline and recovery of coral cover. This is concerning as the constant loss of adults undercuts overall reproductive potential (Hughes and Tanner 2000).

These previous results underscore the critical need for fundamental information on coral reproduction, including estimates and trends over time of total fecundity, recruitment, and survival. These results may also suggest that targeting specific, highly fecund species for conservation at local levels may not scale up regionally. Conservation efforts should try to prioritize protecting highly fecund species across multiple levels, irrespective of growth forms, to maximize recovery (Mumby et al. 2011). Targeted conservation focusing on reproductive hotspots within coral species and genera that can enhance reef resilience in the face of climate change and habitat loss should assist in ensuring the long‐term persistence of coral reefs (Bellwood et al. 2004; Quigley 2024).

## Author Contributions

**M. M. Bilodeau:** writing – original draft (equal). **K. M. Quigley:** writing – original draft (equal).

## Funding

This work was supported by Australian Research Council (ARC) DECRA Fellowship to K.M.Q (DE230100284).

## Conflicts of Interest

The authors declare no conflicts of interest.

## Supporting information


**Figure S1:** Comparison between measured fecundity to imputed fecundity across six species. The dashed line represents a 1:1 line, in which any points above the line represented imputed values that are overestimated, and anything below the line represents values that are underestimated.
**Figure S2:** Colony‐level log mean fecundity estimates across coral family. Genera are ordered from highest to lowest median value.
**Figure S3:** Relationship between fecundity per cm^2^ and Indo‐Pacific fecundity across coral genera. The black line represents the linear correlation across all data points. Colors are for each growth form and each point is a coral species.
**Figure S4:** Mean fecundity per cm^2^ per species relative to mean fecundity per genus. Gray dots and the dashed black lines represent the mean fecundity per cm^2^ per genus. Species with a mean value greater than the mean per genus are colored in green and those below the average are colored in red.
**Table S1:** Growth form correlations for fecundity per cm^2^.

## Data Availability

The data and code supporting the results of this study are archived in a Zenodo repository and are available via the following link https://zenodo.org/records/19364002.
