# Supplementary material for: Patterns in Coral Fecundity Across the Indo‐Pacific
Source: Ecol Evol. 2026 Oct 2;16(10):e74441. doi: 10.1002/ece3.74441 (PMC13633411; doi:10.1002/ece3.74441)
Supplement: Supplementary file 1 [file ECE3-16-e74441-s001.docx]

**Supplementary Figures**

**Supplementary Figure 1.** Comparison between measured fecundity to imputed fecundity across six species. The dashed line represents a 1:1 line, in which any points above the line represented imputed values that are overestimated, and anything below the line represents values that are underestimated.

**Supplementary Figure 2.** Colony-level log mean fecundity estimates across coral family. Genera are ordered from highest to lowest median value.

**Supplementary Figure 3.** Relationship between fecundity per cm² and Indo-Pacific fecundity across coral genera. The black line represents the linear correlation across all data points. Colours are for each growth form and each point is a coral species.

**Supplementary Figure 4.** Mean fecundity per cm^2^ per species relative to mean fecundity per genus. Grey dots and the dashed black lines represent the mean fecundity per cm^2^ per genus. Species with a mean value greater than the mean per genus are coloured in green and those below the average are coloured in red.

**Supplementary Tables**

**Supplementary Table 1:** Growth form correlations for fecundity per cm^2^

| **Growth Form** | **p value** | **R^2^** |
| --- | --- | --- |
| Massive | 0.501 | 0.0068 |
| Branching | 0.355 | 0.0179 |
| Laminar | 0.0385 | 0.1235 |
| Encrusting | 0.0406 | 0.1284 |
| Digitate | 0.582 | 0.0172 |
| Other | 0.104 | 0.0384 |
